# Psychotherapy and brain plasticity

**DOI:** 10.3389/fpsyg.2013.00548

**Published:** 2013-09-06

**Authors:** Daniel Collerton

**Affiliations:** ^1^Clinical Psychology, Northumberland, Tyne and Wear NHS Foundation TrustGateshead, UK; ^2^Newcastle upon TyneNewcastle University, UK

**Keywords:** plasticity, psychotherapy, functional magnetic resonance imaging, multivoxel pattern analysis, emotion, consciousness

## Abstract

In this paper, I will review why psychotherapy is relevant to the question of how consciousness relates to brain plasticity. A great deal of the research and theorizing on consciousness and the brain, including my own on hallucinations for example (Collerton and Perry, 2011) has focused upon specific changes in conscious content which can be related to temporal changes in restricted brain systems. I will argue that psychotherapy, in contrast, allows only a focus on holistic aspects of consciousness; an emphasis which may usefully complement what can be learnt from more specific methodologies.

## Introduction

For the last century or so, psychotherapy has aimed to change the mind. And if it has been effective in doing so, it must have lead to lasting changes in conscious content, and potentially in the process of consciousness itself. Few other human endeavors seek so systematically to produce predictable enduring variation in emotion, cognition, behavior and somatic perceptions; changes which can persist for many years beyond the end of therapy. Over the last few decades, as methods have become available for measuring brain structure and function, it has provided a potential real-life method by which meaningful changes in consciousness can be related to measures of brain function. Admittedly, thus far interest has been on understanding what brain function can tell us about how psychotherapy works (for example, Jokić-Begić, 2010) rather than the aim of this paper—what psychotherapy can tell us about how the brain and mind are linked. However, in theory, it should be possible to assess consciousness, or at least some aspects of it, before and after psychotherapy, and to relate these to brain changes. And indeed, as described later, such studies have been done. The evidence is still sporadic and somewhat contradictory, but there is more potential now than ever before to correlate psychotherapy-related changes in mind to changes in brain.

In this paper, I will survey the current situation, outlining the formidable conceptual and practical difficulties which still need to be overcome, and suggest a potential strength of psychotherapy as a tool for understanding consciousness which, in combination with advances in functional imaging analysis, may give a way forwards.

## Challenges of definition and measurement

There are a number of intractable definitional and measurement issues in this field which have been only partially solved.

Consciousness itself is a fuzzy concept—and its components are no clearer, as illustrated by the continuing discussions on whether it even exists as a meaningful phenomenon. James explicitly posed the question in the first, 1904, volume of the Journal of Philosophy, Psychology, and Scientific Methods and over a century later the answer is still not clear. As exemplified by Newell and Shanks (in press), the nature and role of consciousness and its relationship to behavior are still under discussion.

The term psychotherapy equally lacks definition. It has been applied to an exceptionally wide range of approaches with differing models, techniques, goals, and outcomes. Feltham and Horton's (2012) Handbook, for example, surveys some 23 major approaches from Dialectical Behavior Therapy to Psychodrama.

Finally, though methods of investigating *in vivo* brain function are incomparably better than even a decade ago, they are still limited in cognitive, temporal, and spatial resolution, while the large and intrusive technology involved limits applications to settings which bear little relationship to everyday life.

In the face of fuzzy concepts and methods, there is a scientific temptation to retreat into investigating relatively specific, easily measureable, aspects of consciousness and the brain. However, looked at in another way, this very lack of focus may be a strength rather than a weakness in that it forces attention to holistic changes in consciousness which can then be related to systemic changes in neural networks.

## The effects of psychotherapy

Within the vast variety of psychotherapies, Cognitive Behavioral Therapy (CBT) has become the most widely accepted approach, and has amassed a strong body of evidence of effectiveness (Butler et al., 2006). It leads to long lasting, reproducible changes in emotion, cognition, behavior, and somatic symptoms across a range of mood and other psychological disorders. This strongly suggests that CBT is a powerful means of changing consciousness. CBT has always had a marked emphasis on measurement and it is also the therapy whose effects have most often been related to brain changes. For these reasons, I will take it as the exemplar psychotherapy for the purpose of this paper, while acknowledging that other approaches may be equally valid.

If we want to relate the effects of psychotherapy on consciousness to brain changes, it would seem necessary to know the changes it produces and how it does so. Consciousness itself is not directly accessible of course, so, as summarized in Table 1, researchers have taken a number of routes to inferring what is in the consciousness of a patient. Self-report, observer report, behavioral measures, and experimental tasks can be used to more or less directly infer changes in the content of patient's conscious thought. Taken together, these methodologies have produced good evidence that the complex combinations of emotions, cognitions, behaviors and somatic symptoms which characterize mood disorders do shift as a result of CBT.

**Table 1:** **Indicators of consciousness used in CBT outcome studies**.

**Type of measure**	**Example measures**	**Illustrative study**
Self-report	Structured questionnaires e.g., Beck Depression Inventory (Beck et al., 1988)	Elkin et al., 1995
Observer report	Structured observer ratings e.g., Hamilton Depression Rating Scale (Bagby et al., 2004)	Teasdale et al., 2000
Behavioral measures	Observable behavioral change e.g., return to employment	Della-Posta and Drummond, 2006
Experimental tasks	Measures of perceptual distortion e.g., body morphing (Benson et al., 1999)	Cornelissen et al., 2013

## What changes as a consequence of psychotherapy?

However, evidence is lacking as to what specifically changes as a consequence of psychotherapy (see, for example, Murphy et al., 2009). Despite the range of different ways of measuring the effects of psychotherapy noted above, it is striking how closely these are related. Thus, change in one symptom area, for example cognition, is accompanied by changes in other symptom areas such as emotion or behavior; at least as averaged over the timescales and group numbers common in treatment trials.

Similarly, though there is evidence that different modalities of therapy may have different levels of effectiveness (see Tolin, 2010 for a meta-analytic comparison of CBT with other therapies), where this does occur this appears to be more a quantitative than a qualitative difference. The outcomes of psychodynamic, person-centred, and behavioral psychotherapy are broadly equivalent despite their varieties of approaches and targets for therapeutic change (Stiles et al., 2008; Budd and Hughes, 2009) perhaps because they work via common final paths (Mansell, 2011).

Even treatments as different as CBT and pharmacotherapy appear to have broadly similar outcomes in, for example, depression (DeRubeis et al., 2008) with no reliable evidence of the differential effects of CBT on negative cognitions or medication on somatic symptoms as might have been expected from their mechanisms of action. Attempts to predict which patients might benefit from any specific intervention have not been successful.

Taking all of these comparisons together, there is very little evidence that specific areas of consciousness can change in a meaningful way without these effects rippling through the rest of consciousness. This suggests that trying to fractionate the effects of psychotherapy on consciousness into components might not be possible. It is the totality of consciousness which changes as a result of CBT rather than one specific aspect of it.

## Implications for linking the effects of psychotherapy to brain plasticity

This has implications for relating the effects of CBT to brain plasticity. Traditionally, neuroscience has adopted a reductionist approach to the brain; relating specific psychological functions to specific brain areas. This has been enormously successful with many psychological functions. Amongst many other pairings, neuropsychological localization has linked learning to the hippocampus and other structures in the medial temporal lobe, object perception to the ventral visual stream, and language to the left temporal cortex. However, the localization paradigm has left in its wake the binding problem—how the functions of disparate brain areas are tied together to produce the usual subjective sense of a single coherent consciousness. In this context, perhaps the holistic effects of CBT and other psychotherapies on consciousness become a means of side stepping the binding problem. If significant changes in bound consciousness can be related to restricted changes in brain function, that might narrow down the candidate brain areas which may underlie consciousness.

## Effects of CBT on measures of brain function

The plasticity in human brain which underlies individual, idiosyncratic and instantaneous elements of consciousness—specific words, thoughts, images, feelings, and memories—comes from tiny, subtle, and dynamic changes which are embedded within and across networks of microscopic cells. In comparison, our ways of measuring plasticity in the human brain have to trade off cognitive (the ability of scans to resolve precise psychological states, particular memories for instance), spatial, and temporal resolutions with even the best resolution vastly greater than the fundamental mechanisms of plasticity. The spatial resolution limit of a Magnetic Resonance Imaging (MRI) scan, our best current means of directly assessing brain structure and function, contains somewhere around 9 million brain cells (deCharms, 2008).

However, this has not prevented functional imaging, particularly functional MRI (fMRI) to identify which areas of the brain change following psychotherapy. (There have been a rather small number of structural imaging studies of the effects of psychotherapy, mainly in eating disorders, but many of these have been confounded by the effects of weight gain and loss on brain structure (Lobera, 2011) making it difficult to interpret their results).

The dominant paradigm has been to compare levels of brain activity pre and post CBT to see what changes. This approach has mainly been used in depression and has identified that changes are localized to specific frontal, cingulate, and limbic areas. There is decreased activity in the limbic system, especially the amygdala, with dorsolateral prefrontal cortex becoming relatively more active and orbitomedial and cingulate cortex less so; a move toward normality from patterns observed before treatment (Ochsner et al., 2002; Goldapple et al., 2004; Malhi et al., 2004; Ritchey et al., 2011; Höflich et al., 2012) and consistent with what is know of the processing of emotional stimuli (Simpson et al., 2000; Northoff et al., 2004; Leppänen, 2006; Beck, 2008). Pre-treatment levels of cingulate activity can even predict response to CBT with some reliability (Konarski et al., 2009; Ritchey et al., 2011; Siegle et al., 2012).

There have not been comparisons between the effects of different types of psychotherapy on the brain (potentially interesting in view of their equivalent effects on consciousness), but there are conflicting reports of the effects of pharmacotherapy; similar changes in brain activity to those following psychotherapy were not seen after antidepressant treatment by Goldapple et al. (2004) though they were identified by Furmark et al. (2002); opening up, but not confirming, the possibility that different brain changes might have similar effects on consciousness.

Taken as a whole, this evidence would suggest that the holistic changes in consciousness seen after psychotherapy for depression are associated with changes in a relatively restricted number of brain areas; mainly frontal, cingulate, and limbic cortex, with the implication that plasticity in those areas is particularly associated with persistent variations in consciousness.

However, a one to one correspondence between change in depression and change in specific brain areas may be over stated (Linden, 2006; Frewen et al., 2008; Dichter et al., 2012). For example, very similar changes in those brain areas are seen after CBT and other psychological treatments for anxiety (Furmark et al., 2002; Paquette et al., 2003; Straube et al., 2006; Porto et al., 2009; Freyer et al., 2011), schizophrenia (Wykes et al., 2002), eating disorders (Vocks et al., 2011) and Irritable Bowel Syndrome (Lackner et al., 2006). This is consistent with a consciousness network which depends upon these brain areas (and no doubt others) but it also suggests that there is a large overlap in the brain changes associated with different holistic states of consciousness. At present, fMRI data would suggest that, simply put, CBT is associated with a decrease in emotionality (less limbic activity) and an increase in thoughtfulness (increased dorsolateral frontal activity), as would be expected from its aims and methods (Clark and Beck, 2010). Though we may be able to link consciousness to a subset of anatomical structures using psychotherapy as an investigative tool, we appear to lack specificity in our account of how different states of consciousness could arise. Is it that in using psychotherapy to localize holistic changes in consciousness to a restricted set of brain structures, we have lost the ability to account for why consciousness is so idiosyncratic and so changeable?

## Apotential way forward

An analogous challenge has arisen in fMRI studies of visual perception. Early attempts to localize specific perceptions to specific brain areas worked only for grossly different stimuli—visualizing navigating a house compared to imagining playing tennis (Owen et al., 2006)—or for simple stimuli in early, highly specialized, visual areas (Kay et al., 2008). More latterly, however, multivoxel pattern analysis (MVPA), in which patterns of activity across wide areas of the brain are analyzed, has produced a significant increase in cognitive resolution. Fairly similar stimuli, for example chairs and shoes (Norman et al., 2006; deCharms, 2008; Poldrack, 2011), or over-riding categories of images such as living or non-living (Naselaris et al., 2012) can now be recognized from pattern information fMRI (Formisano and Kriegeskorte, 2012) data. Not only perceptions, but also images and memories (Chadwick et al., 2012; Rissman and Wagner, 2012) are starting to be distinguished. Beginnings are starting to be made in reproducing data across as well as within subjects (Accamma and Suma, 2012; Raizada and Connolly, 2012). Significantly, cognitive resolution appears to increase as the focus of the analysis is widened to include more brain areas.

MVPA might therefore lead to the ability to map holistic changes in consciousness to patterns within and across the regions that classic fMRI has identified as responsive to psychotherapy (Siegle et al., 2007). Thus it may provide the mechanism to bridge holistic and specific variations in consciousness and brain.

## Conclusions

It is clear that CBT, and probably other psychotherapies, alters consciousness in personally important, lasting, and measurable ways. Brain function and brain structure are different after CBT. Looking at functional changes in the brain suggests that consciousness changes in response to plasticity in the linked systems of the frontal, cingulate, and limbic cortices. However, we do not know how modulations in those areas link to different states of consciousness. Using the most recent imaging analysis to map activity simultaneously across these regions might give the missing specificity; allowing whole brain changes to be mapped to holistic changes in consciousness.

In order to do this, our next challenge will be to develop ways of capturing the experience of consciousness as a whole rather than, as we have tried to do in the past, the individual thoughts, images, and emotions which are bound together to produce it.

### Conflict of interest statement

The author declares that the research was conducted in the absence of any commercial or financial relationships that could be construed as a potential conflict of interest.
